# Human Leukocyte Antigen as a Key Factor in Preventing Dementia and Associated Apolipoprotein E4 Risk

**DOI:** 10.3389/fnagi.2019.00082

**Published:** 2019-04-12

**Authors:** Lisa M. James, Apostolos P. Georgopoulos

**Affiliations:** ^1^Department of Veterans Affairs Health Care System, Brain Sciences Center, Minneapolis, MN, United States; ^2^Department of Neuroscience, University of Minnesota Medical School, Minneapolis, MN, United States; ^3^Department of Psychiatry, University of Minnesota Medical School, Minneapolis, MN, United States; ^4^Department of Neurology, University of Minnesota Medical School, Minneapolis, MN, United States

**Keywords:** human leukocyte antigen (HLA), herpes virus, Alzheimer's disease, apolipoprotein E cognition, brain

Itzhaki's (2018) recent review discusses the evidence for a role of herpes virus (mainly herpes virus 1) in the development of Alzheimer's disease (AD), particularly among genetically vulnerable individuals. Specifically, the viral concept proposes that latent herpes virus in the brain of apolipoprotein E4 (apoE4) carriers is intermittently reactivated causing cumulative damage that ultimately results in AD. The viral concept and collective findings are particularly intriguing given the potential for intervention for AD aimed at neutralizing or eliminating herpes virus. Here we discuss human leukocyte antigen (HLA) as an additional genetic link in the viral concept of AD that not only accounts for the role of herpes virus in AD, but also extends to other viruses that may contribute to AD and to other diseases, and is consistent with beneficial brain effects of treatments aimed at eliminating the damaging effects of herpes virus via antivirals or IVIG as discussed in the review.

Human leukocyte antigen (HLA) genes play a critical role in immune protection from foreign antigens including viruses, bacteria, and parasites (Meuer et al., 1982). HLA genes, which are the most highly polymorphic in the human genome, orchestrate production of cell-surface glycoproteins that facilitate immune surveillance and initiate an immune response to eliminate cytosolic or extracellular foreign antigens through cell destruction or antibody production. Recent studies have implicated the HLA region in neurodegenerative diseases including AD (Lambert et al., 2013; Steele et al., 2017); however, the concept of HLA-disease associations runs counter to the biological and evolutionary role of HLA which is to protect against invaders and promote species survival. Indeed, the literature is replete with studies highlighting the protective role of HLA in various conditions. For example, HLA-DRB1^*^13:02 has been shown to confer protection against various illnesses ranging from hepatitis B and C (Singh et al., 2007), influenza (Posteraro et al., 2014), HIV (Pereyra et al., 2010), malaria (Hill et al., 1991), and numerous autoimmune disorders (Bettencourt et al., 2015). Perhaps the most relevant in this case is recent evidence of HLA-protection against structural and functional age-related brain changes (James et al., 2018a,b). Notably, DRB1^*^13:02, has been shown to not only protect against age-related deterioration in neural network functioning, but also to negate the deleterious effects of apoE4 on neural network functioning (James et al., 2018b; Figure 1, top), suggesting a common pathway.

**Figure 1:**
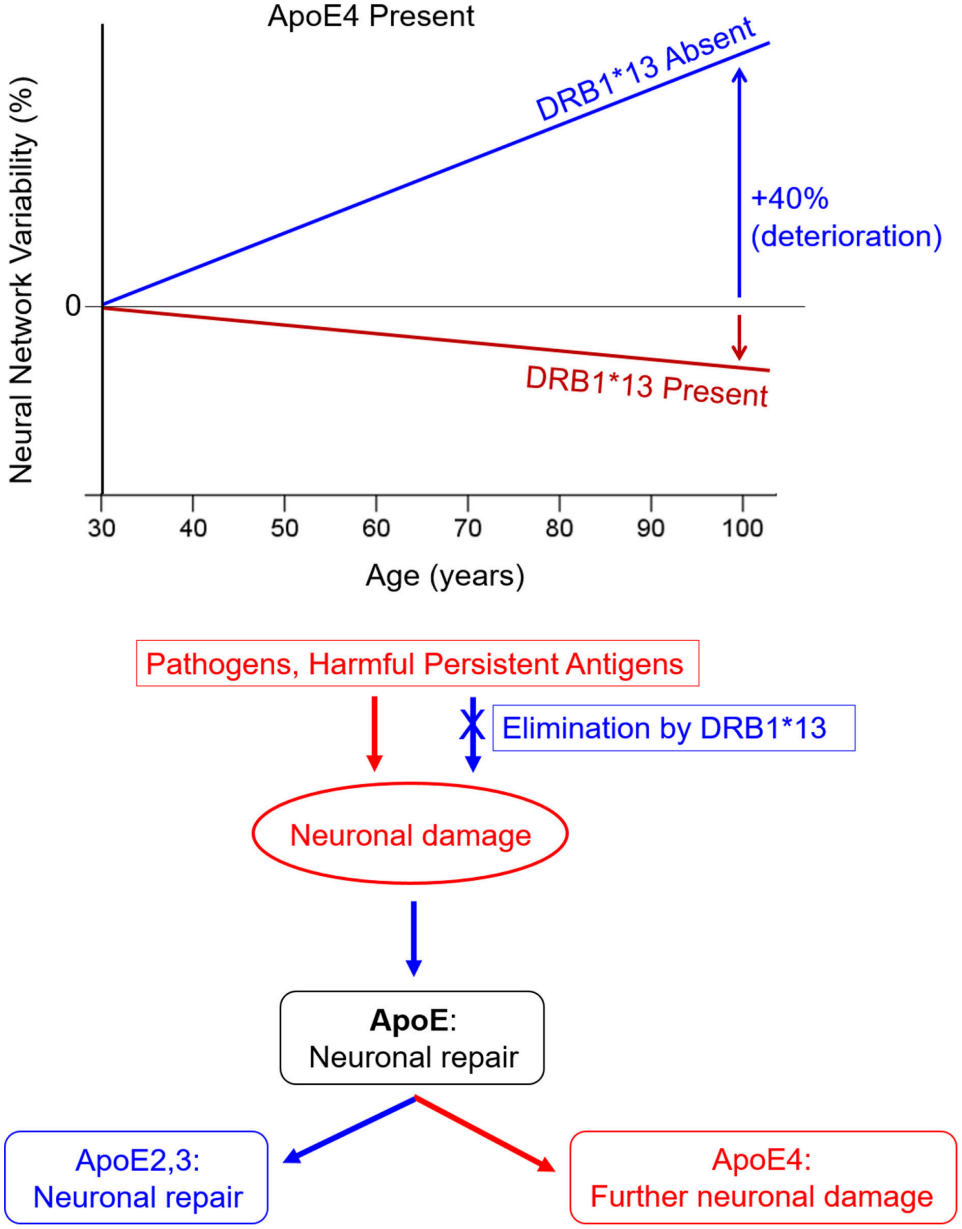
HLA DRB1*13 and ApoE effects on brain health. **(Top)** HLA protection against age-related increases in neural network variability in apoE4 carriers (adapted from James et al., 2018b). **(Bottom)** Hypothesized neuroimmune cascade in which persistent antigens cause initial neuronal damage, stimulating apoE synthesis. Expression of neurotoxic apoE4 causes further damage whereas expression of apoE2 or apoE3 facilitates neuronal repair.

Successful elimination of viruses and other foreign antigens hinges on a match between HLA and epitopes derived from foreign antigen proteins. A search of the Immune Epitope Database (www.iedb.org) indicates that 78 human herpes virus 1 epitopes match with HLA class I proteins and 72 epitopes match with HLA class II proteins. Furthermore, 23 human herpes virus 1 epitopes were found to bind with DRB1^*^13:02, the same genes that we have found to protect against age-related brain changes (James et al., 2018a,b), with high affinity using the Sturniolo et al. (1999) method. These results support the hypothesis that several HLA proteins are effective for elimination of human herpes virus 1 either through apoptosis (Class I) or antibody production (Class II). Similar IEDB searches indicate that DRB1^*^13:02 binds with epitopes from several other common pathogens including influenza (both hemagglutinin and neuraminidase), alphapapillomavirus, hepatitis B and C, HIV, mycobacterium tuberculosis, plasmodium falciparum, and plasmodium vivax (malaria parasites), yellow fever, and various types of mammarenavirus, indicative of ability to produce antibodies to eliminate these foreign antigens. However, each individual has a limited repertoire of HLA genes, some of which may not be able to successfully eliminate foreign antigens due to HLA-antigen incongruence. In the absence of a match, the antigens may persist leading to inflammation, cell damage, and autoimmunity (Institute of Medicine National Research Council, 2010). Notably, inflammation is a well-known contributor to AD pathogenesis (Akiyama et al., 2000), underscoring the likely involvement of immune-mediated genes such as HLA in AD risk and protection. In terms of the viral concept of AD, the Persistent Antigen hypothesis (James et al., 2017; James and Georgopoulos, 2018) suggests that individuals who are unable to sufficiently eliminate herpes virus due to HLA-antigen incongruence will experience immunoregulatory disturbances and may ultimately be at risk of developing degenerative diseases including AD. Indeed, any foreign antigens including other viruses, bacteria, and parasites that are insufficiently eliminated may lead to a similar fate. That is, it is lack of protection inherent in an HLA-antigen mismatch that underlies HLA-disease associations including AD rather than HLA polymorphisms themselves conferring risk. To that end, we propose that an HLA-antigen mismatch initiates a neuroimmune cascade, the outcome of which depends on apoE genotype as follows (Figure 1, bottom): (1) an HLA-antigen mismatch results in the persistence of antigens which directly cause neuronal damage and inflammation; (2) apoE is synthesized in response to neuronal damage to facilitate repair (Mahley and Huang, 2012); (3) neuronal expression of apoE2 or apoE3 support neuronal repair; however, expression of apoE4 results in neurotoxic fragments (due to protein instability resulting from domain interactions) which cause mitochondrial dysfunction and cytoskeletal alterations that ultimately contribute to neurodegeneration (Mahley and Huang, 2012). Alternatively, a match with sufficiently high affinity between one's HLA composition and foreign epitopes enables antibody production and elimination of the antigen, preventing the subsequent neuroimmune cascade.

Although additional research is needed to corroborate the essential role of HLA and persistent antigens in the viral concept of dementia, support for the critical role of HLA in disease outcomes is offered by recent studies on Gulf War Illness (GWI), a condition that, like AD, is associated with neurocognitive and mood disturbances in addition to symptoms affecting multiple peripheral systems. First, we found that 6 HLA alleles, including DRB1^*^13:02, distinguished healthy and affected veterans (Georgopoulos et al., 2016). Specifically, GWI cases, but not healthy veterans, lacked the 6 HLA alleles suggesting a genetic vulnerability that hindered clearance of foreign antigens associated with that period of service. Second, although significant brain atrophy has been observed in GWI (Christova et al., 2017), HLA-DRB1^*^13:02 has been shown to protect against brain atrophy in Gulf War veterans (James et al., 2017). Finally, the addition of GWI serum to neural cultures has been shown to result in neural network disruption, apoptosis, and reduced spreading (Georgopoulos et al., 2018), indicative of the deleterious effects of circulating GWI-related pathogens on the brain. However, those detrimental effects were reduced with the addition of serum from healthy veterans (who possessed protective HLA alleles) (Georgopoulos et al., 2018) or immunoglobulin G (IgG) to the cultures (Tsilibary et al., 2018), both of which presumably neutralize or eliminate circulating pathogens. We suspect a similar mechanism underlies the beneficial effects of IVIG and antivirals as it relates to herpes virus and AD discussed in the review.

Here we offer a novel perspective on the viral concept of AD with HLA playing a pivotal early role. From this perspective the HLA-antigen match is fundamental in health maintenance whereas an HLA-antigen mismatch plays a primary role in disease etiology via circulation of persistent antigens. Therefore, elimination of persistent antigens via personalized precision immunotherapy is indicated as a potentially beneficial intervention for AD and other immune-implicated conditions. Such work is underway in our laboratory.

## Author Contributions

All authors listed have made a substantial, direct and intellectual contribution to the work, and approved it for publication.

### Conflict of Interest Statement

The authors declare that the research was conducted in the absence of any commercial or financial relationships that could be construed as a potential conflict of interest.
